# Identification and functional characterization of the Piezo1 channel pore domain

**DOI:** 10.1074/jbc.RA120.015905

**Published:** 2020-12-29

**Authors:** Elena. D. Nosyreva, David Thompson, Ruhma Syeda

**Affiliations:** Department of Neuroscience, University of Texas Southwestern Medical Center, Dallas, Texas, USA

**Keywords:** mechanosensitive ion channels, pore domain, mechanically activated Piezo1, single channel recordings, patch clamp, channel activation, permeation, mechanotransduction, single-molecule biophysics, DMEM, Dulbecco’s modified Eagle’s medium, I-V, current–voltage

## Abstract

Mechanotransduction is the process by which cells convert physical forces into electrochemical responses. On a molecular scale, these forces are detected by mechanically activated ion channels, which constitute the basis for hearing, touch, pain, cold, and heat sensation, among other physiological processes. Exciting high-resolution structural details of these channels are currently emerging that will eventually allow us to delineate the molecular determinants of gating and ion permeation. However, our structural–functional understanding across the family remains limited. Piezo1 is one of the largest and least understood of these channels, with various structurally identified features within its trimeric assembly. This study seeks to determine the modularity and function of Piezo1 channels by constructing deletion proteins guided by cryo EM structural knowledge. Our comprehensive functional study identified, for the first time, the minimal amino acid sequence of the full-length Piezo1 that can fold and function as the channel’s pore domain between E2172 and the last residue E2547. While the addition of an anchor region has no effect on permeation properties. The Piezo1 pore domain is not pressure-sensitive and the appending of Piezo Repeat-A did not restore pressure-dependent gating, hence the sensing module must exist between residues 1 to 1952. Our efforts delineating the permeation and gating regions within this complex ion channel have implications in identifying small molecules that exclusively regulate the activity of the channel’s pore module to influence mechanotransduction and downstream processes.

Nearly all structures in our body experience mechanical forces, from large-scale organs down to the cellular level. On a molecular scale, these forces are detected by ion channels (1, 2), G protein-coupled receptors (3, 4), primary cilia (5, 6), and glycocalyx (7), all of which function as mechanotransducers, converting physical forces into electrochemical responses. Piezos are the first cloned excitatory mechanically activated ion channels identified in vertebrates and are responsible for mechanotransduction in both neuronal and nonneuronal cells (8). Since discovery in 2010, significant efforts have elucidated the physiological roles of the Piezo family, which consists of Piezo1 and Piezo2. Piezo channels are now well established as critical sensors of touch and pain (9), volume regulation (10), shear stress (11), baroreception (12), and proprioception (13). They play crucial roles in respiratory physiology (14), and likely participate in other important functions yet to be discovered. Their dysfunction is linked to diverse pathologies including lymphedema (15), xerocytosis (16), tactile pain (17), and somatosensory disorders (18).

Mechanical forces such as shear stress, pressure, and membrane tension are shown to activate Piezo1 channels (19, 20, 21, 22) and lead to the overall understanding of mechanosensitive gating. However, the structural determinants of ion permeation and pressure sensitivity are not fully understood. Piezo1 is a pore-forming subunit of mechanically activated ion channels that follows the force-from-lipid gating paradigm, signifying that it harbors both the ion-conducting pore and mechanosensitive elements within its 2547 amino acid polypeptide (2, 21). The identified biophysical characteristics of Piezo1 include inactivation that also has an element of voltage dependence (23, 24, 25), outward rectification (2, 8), along with current block by ruthenium red and toxin peptide GsMTx4 (26, 27). This study is guided by the fundamental tenet that mechanosensitive Piezo1 is modular in design, and that the coupling between the component modules underlies their pressure sensitivity and functional diversity. As seen in numerous voltage-gated sodium and potassium channels (28, 29, 30, 31), there is a distinct pore domain that is conductive when it is devoid of its voltage-sensing module. In an intact protein, the opening and closing of the pores are tightly controlled by the voltage-sensing module so that it generates electrical signals only upon stimulus. Here, we seek to discover whether Piezo1 also harbors a discrete pore domain that can express and fold, while remaining functional without the sensing module.

Piezos are the largest family of plasma membrane ion channels identified to date. They are nonselective cation channels that assemble as a homotrimer, totaling ∼7500 amino acids and 114 transmembrane helices. The high-resolution cryogenic electron microscopy revealed fascinating structural features (32, 33, 34), such as an extracellular cap domain, an intracellular beam, and nine repetitive structural motifs that constitute the basis of its curved blade shape. Ion permeation likely occurs through a central pore, which is lined by the last two transmembrane helices and an intracellular C-terminal domain. In all the solved structures, the tightest constriction is too narrow to allow cationic flow, and the pore radius at constriction site P2536/E2537 is the lowest at ∼4 Å. Hence, currently available Piezo1 structures are presumed to represent a closed conformation.

Here we used a series of deletion mutant channels to identify the minimal pore domain of the Piezo1 channels, hereby addressing hypothesis-driven questions: i) Is there a canonical pore domain in Piezo1 that is conductive without the sensing module? ii) Is the putative pore intrinsically open, or is there an element of pressure sensitivity retained in the pore domain? To address these questions, our strategy involved utilizing the heterologous expression of the putative pore domains in Piezo1 knockout HEK cells, followed by a comprehensive single-channel functional characterization to extensively study ion permeation, gating, and pharmacological properties.

## Results

### Identification of Piezo1 pore domain

**Figure 1:**
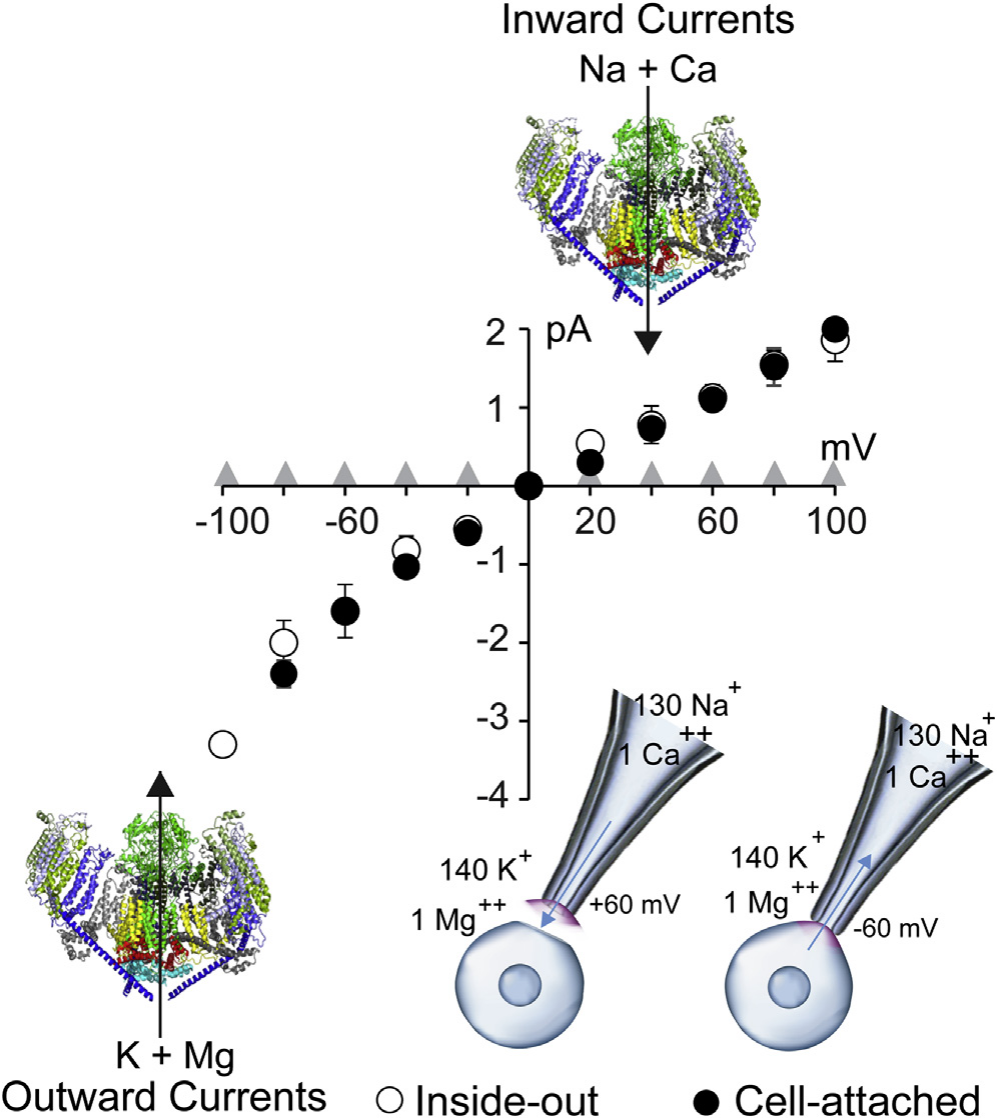
**Piezo1 single-channel currents in various patch-clamp modes.** The single-channel current–voltage comparison of full-length Piezo1 elicited by −15 mmHg, separately acquired in an inside-out (n ≥ 5) or cell-attached mode (n ≥ 5), as shown in an *inset*. At positive potentials inward currents are carried by Na^+^ and Ca^++^ in the pipette solution (shown by an *arrow*). At negative potentials outward currents are carried by the bath solution (K^+^ and Mg^++^, in an inside-out mode) or cellular ionic conditions (predominantly K^+^, in a cell-attached mode). *Gray triangles* indicate recordings from HEK cells transfected with empty expression vector pcDNA3.1 (n = 6).

In this study, we performed inside-out patch clamp recordings to control the ionic conditions with respect to the intracellular and extracellular side of the channel. This is in contrast with most of the single-channel Piezo1 data in the literature, which is typically acquired *via* cell-attached electrophysiology (2, 35, 36, 37). Therefore, we first compared the single-channel permeation properties of full-length Piezo1 in the cell-attached *versus* inside-out patch configuration. As expected, there were no statistical differences in single-channel currents when tested between −100 mV and +100 mV in the presence of negative applied pressure (Fig. 1). The slope conductances of Piezo1 calculated from the inside-out and cell-attached data were similar: γ = 14.3 ± 1.4 pS and γ = 15.3 ± 0.2 pS, respectively (n ≥ 9). As a negative control, transfection of empty expression vector (pcDNA 3.1) without the Piezo1 gene did not produce any channel activity (Fig. 1). The outward currents (K+ and Mg++ flow from the intracellular to the extracellular side of the channel) were acquired at negative potentials, and inward currents (Na+ and Ca++ flow from the extracellular to the intracellular side of the channel) were acquired at positive potentials (Fig. 1). These electrical current conventions were used throughout this study.

**Figure 2:**
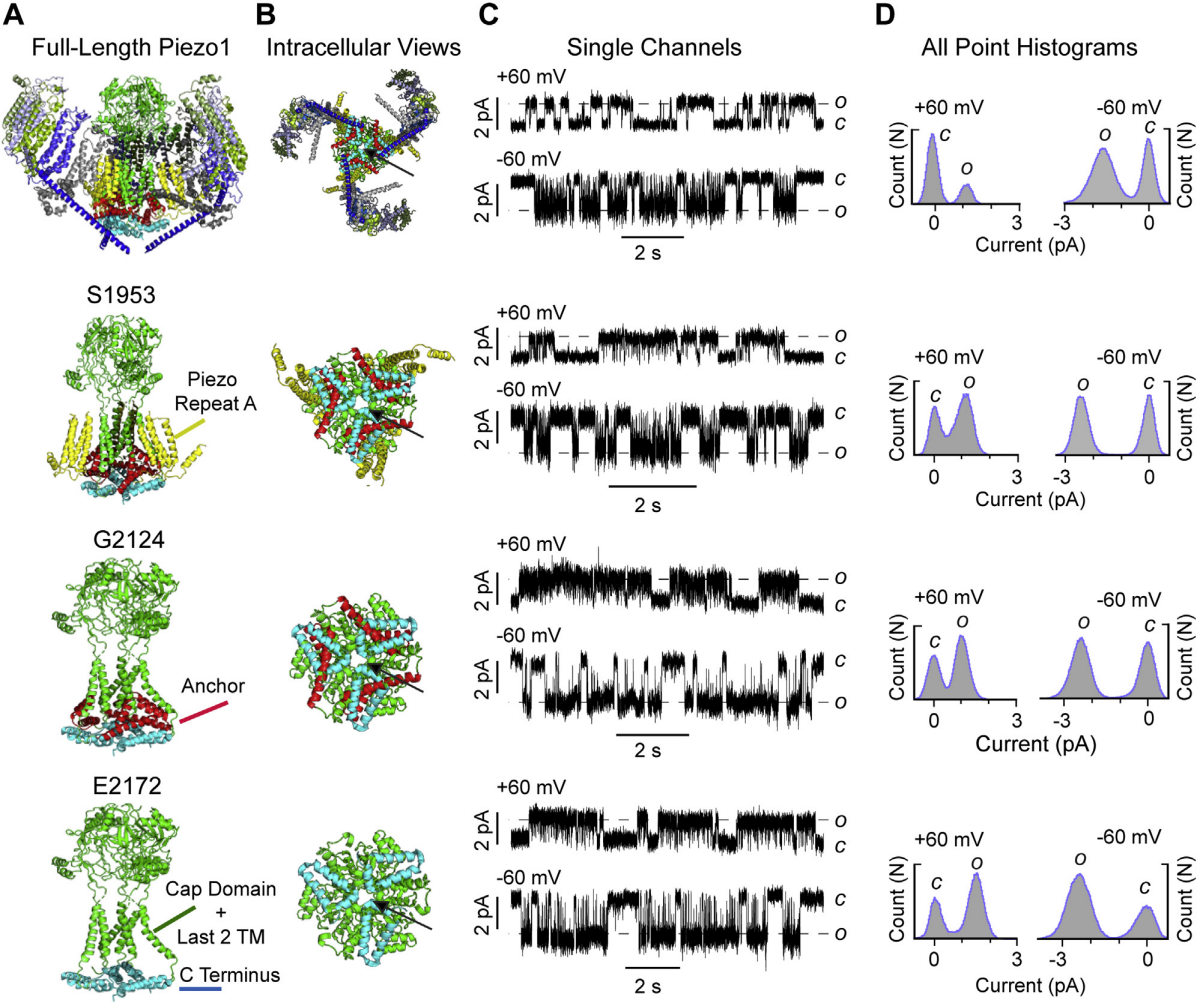
**Identification of Piezo1 pore domain.***A*, the structure of full-length Piezo1 and three putative pore constructs based on cryo-EM data (PDB: 6B3R). The numbers S1953, G2124, and E2172 correspond to the N-terminus starting point and the first amino acid of the truncated proteins. The side views of full-length Piezo1 and the putative pores are shown in the trimeric form from top to bottom. *B*, the intracellular views of full-length Piezo1 and the truncated proteins highlighting the extent of deletions while preserving the permeation pathway (shown by an *arrow*). *C*, single-channel current recordings of the representative Piezo1 and deletion proteins obtained from inside-out patches at +60 mV (*upper*) and at −60 mV (*below*) at −15 mm Hg pressure. *O* and *C* denote open and closed state of the channel. The pipette solution contained 130 mM Na^+^ and 1 mM Ca^++^, the bath solution contained 140 mM K^+^ and 1 mM Mg^++^. *D*, all-point current histograms of the single-channel recordings shown in the panel C. The y-axis represents number of counts either in an open state or closed state that ranges from 10,000 to 25,000 counts.

A Piezo1 cryo-EM structure (PDB: 6B3R) was used as a guide to generate the different truncations of the Piezo1 channels, where ∼1950 amino acids of the N-terminus region were deleted. The selection of three putative pore domains of varying lengths was made to include structurally identified elements while keeping the putative central permeation pathway intact, as suggested by structural studies (32, 33). The longest pore construct started from residue S1953 and contained Piezo Repeat A, the anchor region, the last two TM helices including the extracellular cap, and the C-terminus end (Fig. 2, *A*–*B*; structural elements are color coded). The next construct started from residue G2124 and contained the anchor region, the last two TM helices, including the extracellular cap and the C-terminus end. The shortest pore construct started from residue E2172 and contained just the last two TM helices including the cap and the C-terminus end. Henceforth, they are referred to as S1953, G2124, and E2172 truncated proteins. Next, the truncated constructs were expressed in Piezo1 knockout HEK293 T cells, to avoid the confounding effect of the endogenous full-length Piezo1 in WT HEK cells. Interestingly, all three truncated proteins yielded reproducible channel activity in an inside-out patch configuration. The serial deletion of the protein yielded larger single-channel currents as compared with the full-length Piezo1 when calculated by all point current amplitude histograms, especially at negative applied potentials (Fig. 2, *C*–*D*).

**Figure 3:**
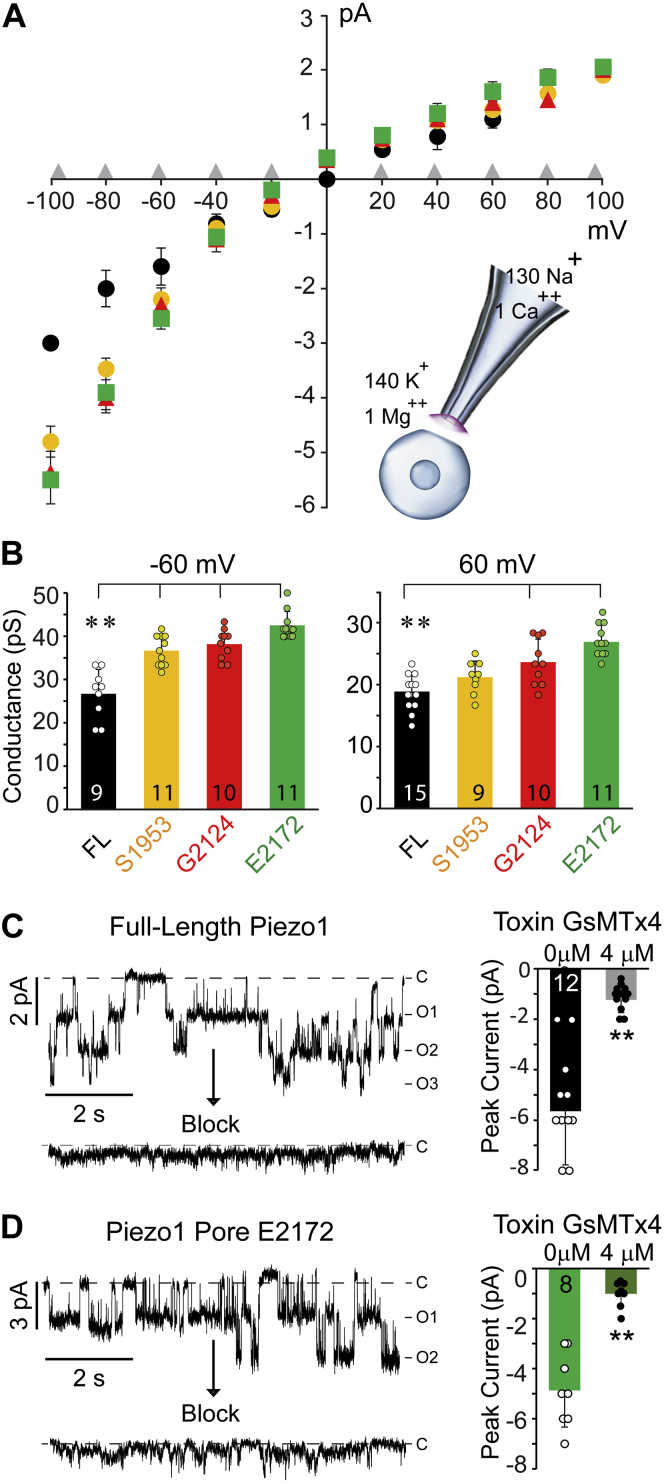
**Voltage-dependent rectification and pharmacology of Piezo1 pore domain.***A*, the current–voltage relationship of full-length Piezo1 (*black*), Piezo1 deletion proteins starting from S1953 (*yellow*), G2124 (*red*), and E2172 (*green*), negative control no-transfection (*gray*) elicited by negative pressure (−15 mmHg). The inset demonstrates the ionic composition of pipette and bath solutions used for all the experiments in an inside-out patch configuration. *B*, comparison of single-channel conductance between full-length Piezo1 and indicated pores at ±60 mV showing voltage-dependent rectification (n ≥ 9). The data and statistics represented as Mean ± Standard Deviation, One-way ANOVA, and two-tailed *t*-test where ∗∗*p* < 0.005. *C*, representative multiple-channel currents of full-length Piezo1 elicited by negative suction in the absence (*upper trace*, n = 12 cells) and presence (*lower trace*, n = 12 cells) of 4 μM extracellular GsMTx-4 toxin, acquired at −60 mV. *D*, representative multiple-channel currents of E2172 pore domain elicited by negative suction in the absence (*upper trace*, n = 8 cells) and presence (*lower trace*, n = 8 cells) of 4 μM extracellular GsMTx-4 toxin, acquired at −60 mV.

The single-channel current–voltage (I-V) plot revealed that the slope conductances (γ) of all deletion proteins were similar to that of full-length Piezo1 (FL γ = 14.3 ± 1.4, S1953 γ = 14.1 ± 0.5 pS, G2124 γ = 15.1 ± 0.2, and E2172 γ = 14.5 ± 0.5 pS) when calculated from the ohmic part of the I-V plots (−40 to +40 mV) (Fig. 3*A*). Conversely, the negative control (transfection of vector) did not yield any channel activity when tested at positive and negative applied potentials (Fig. 3*A*).

Additionally, truncated proteins retained the voltage-dependent rectification, just like the parent full-length Piezo1, suggesting that the permeation pathway and pore properties were intact despite the significant deletion of the rest of the protein. When S1953 was compared with full-length Piezo1, significant difference was found in single-channel conductance at higher potentials (±60 mV onward) (Fig. 3*B*, yellow bars *versus* black bars). Similarly, single-channel conductances of G2124 and E2172 were found to be significantly different from that of full-length Piezo1 at higher negative and positive potentials (Fig. 3*B*, red and green bars *versus* black bars). The T-test and one-way ANOVA analysis led us to conclude the following: i) S1953, G2124 and E2172 channels exhibit similar slope conductance and voltage-dependent rectification, to that of full-length Piezo1. ii) Presence of the anchor region in G2124 has no significant effect on the permeation properties, because E2172 and G2124 exhibit similar steady-state single-channel currents when tested at various potentials.

To further validate that the ionic currents are recorded from Piezo1 and its putative pore, we performed blocking experiments by supplementing 4 μM toxin toward the extracellular side. The peptide toxin GsMTx-4 (derived from Chilean rose tarantula venom) is known to block Piezo1 channels in excised patches, as well as the whole cell mechanosensitive currents evoked by Piezo1 in HEK cells (26, 27). The toxin peptide inhibited both the full-length Piezo1 (n = 12) and the E2172 pore domain (n = 8) currents (Fig. 2, *C*–*D*). The peak current levels decreased ∼fourfold with a ∼75% inhibition in the presence of the toxin for full-length Piezo1 and ∼65% inhibition for pore domain. Taken together, we propose that E2172 is the identified pore domain of Piezo1 that recapitulates single-channel permeation and pharmacological properties of the full-length channels.

### Ion permeation through full-length Piezo1 and pore domain

**Figure 4:**
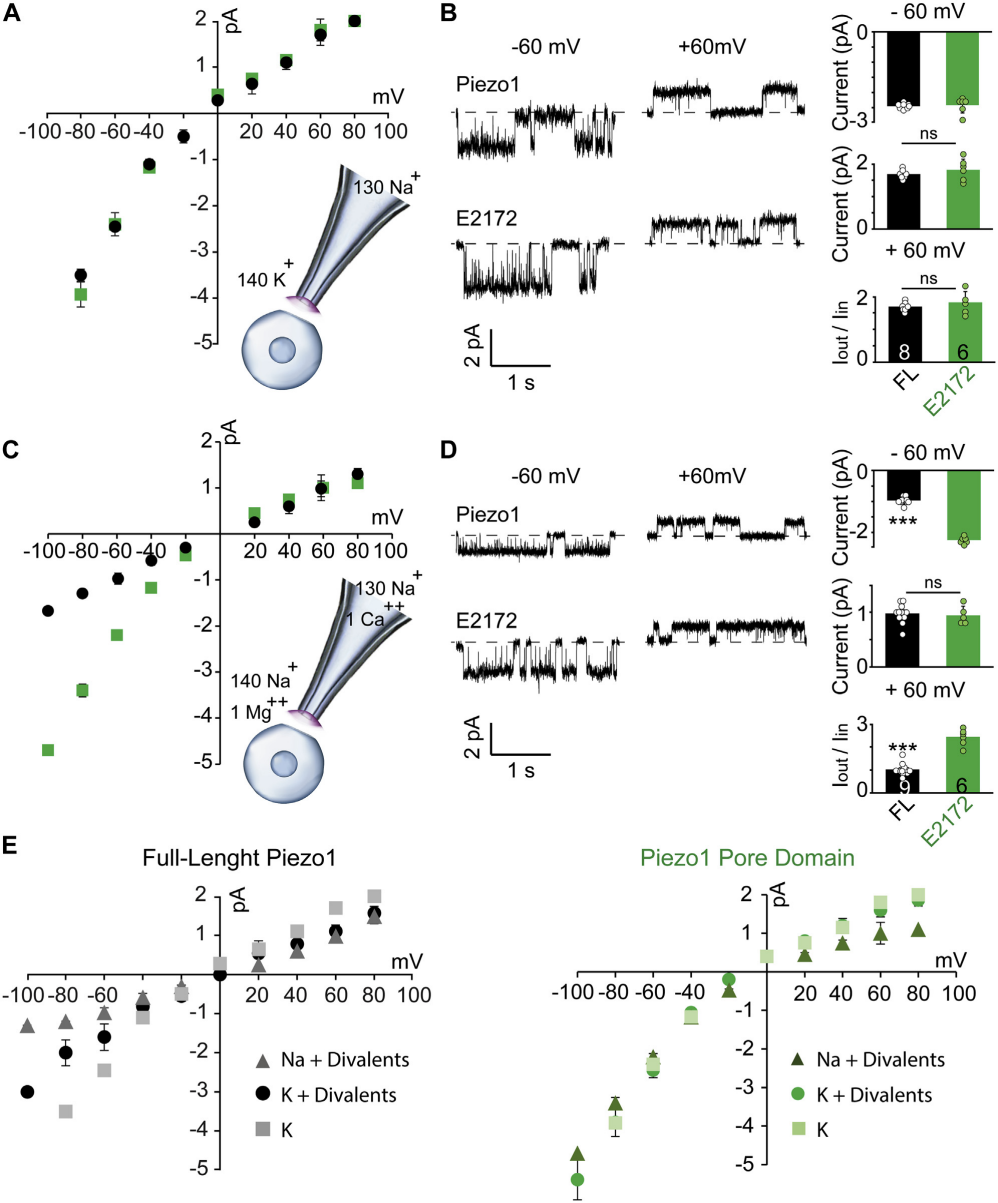
**Permeation properties of full-length Piezo1 and pore domain.***A* and *C*, the current–voltage relationships of full-length Piezo1 (*black*) and Piezo1 pore domain E2172 (*green*), The *inset* demonstrates ionic conditions to acquire data in an inside-out patch configuration: pipette and bath solutions do not contain divalent ions (n ≥ 5) (*A*), pipette and bath solutions contain Na^+^ with divalent ions (n ≥ 5) (*C*). *B*, *left panel*, single-channel current recordings in divalent-free solutions at ±60 mV. The dashed line represents closed state of the channel in each trace. *B*, *right panel*, single-channel current bar graphs at −60 mV (*top*), +60 mV (*middle*), and the I_out_/I_in_ ratio at −60 mV/+60 mV (*bottom*). *D*, *left panel*, single-channel current recordings in symmetric Na+ solutions at ±60 mV. The dashed line represents closed state of the channel in each trace. *D*, *right panel*, single-channel current bar graphs at −60 mV (*top*), +60 mV (*middle*), and the I_out_/I_in_ ratio at −60 mV/+60 mV (*bottom*). The data and statistics represented as Mean ± Standard Deviation, two-tailed *t*-test where ∗∗∗*p* < 0.0005. *E*, the single-channel current–voltage comparison of full-length Piezo1 and E2172 pore domain in various ionic conditions of the bath solution. The currents at each potential is represented as Mean ± Standard Deviation, where n > 6 experiments.

Since Piezo1 is a nonselective cation channel, we determined ion permeation through the parent and deletion proteins in the presence of monovalent and divalent ionic conditions, which have been extensively used in the literature (Figure 1, Figure 2, Figure 3). However, in a multi-ion solution, unfavorable interactions between ions can slow permeation and conductance. The permeation also depends on ion accessibility to the channel’s vestibule and/or permeation pathway. To uncover whether Piezo1 permeation in a multi-ion solution is decreased due to competing interactions, or if there is an ion accessibility issue, we removed divalent ions both from the pipette and bath solution to acquire single-channel data in the presence of monovalent cations (Na^+^ in the pipette, K^+^ in the bath) (Fig. 4*A*). No significant difference in single-channel currents was found between full-length Piezo1 and the pore domain under monovalent ionic conditions. Single-channel currents of full-length Piezo1 increased and surprisingly reached the levels of E2172; both at positive and negative potentials (Fig. 4, *A*–*B*). The slope conductance of the pore domain was equal within error to that of the full-length Piezo1 (FL γ = 15.1 ± 0.6 and E2172 γ = 15.6 ± 0.3 pS) (n ≥ 6) when calculated from the ohmic part of the I-V plots (between −40 and +40 mV). To further assess the contribution of divalent ions in decreasing the conductance, we tested the channel properties in their presence, but changed the bath solution from K^+^ to Na^+^ (Fig. 4, *C*–*D*). As expected, full-length currents at negative potentials decreased in the presence of divalent ions, but not that of the pore domain (Fig. 4*D*). Surprisingly, the voltage-dependent rectification (I_out_/I_in_) of full-length Piezo1 was significantly altered based on the ionic species available for outward currents (monovalent *versus* divalent in the bath solution) (Fig. 4, *D*–*E*).

### Pressure dependent openings of full-length Piezo1 and pore domain

Piezos are mechanically activated ion channels and their gating (transition from closed to open state) depends on physical forces such as pressure, tension, and shear stress. Many point mutations have been identified in various parts of the protein that alter the gating and inactivation properties (34, 36). Here, we sought to determine whether the Piezo1 pore domain E2172 and S1953 deletion mutant (containing Piezo repeat A) encode for any pressure sensitivity. To address this, we compared the pressure-dependent open probability of full-length Piezo1 and pore domain at a single molecule level. The single-channel data has the advantage of directly calculating the channel’s open probability (the time it spends in an open state *versus* the total time of recording trial) without the confounding factors of multiple channels and whole-cell currents.

**Figure 5:**
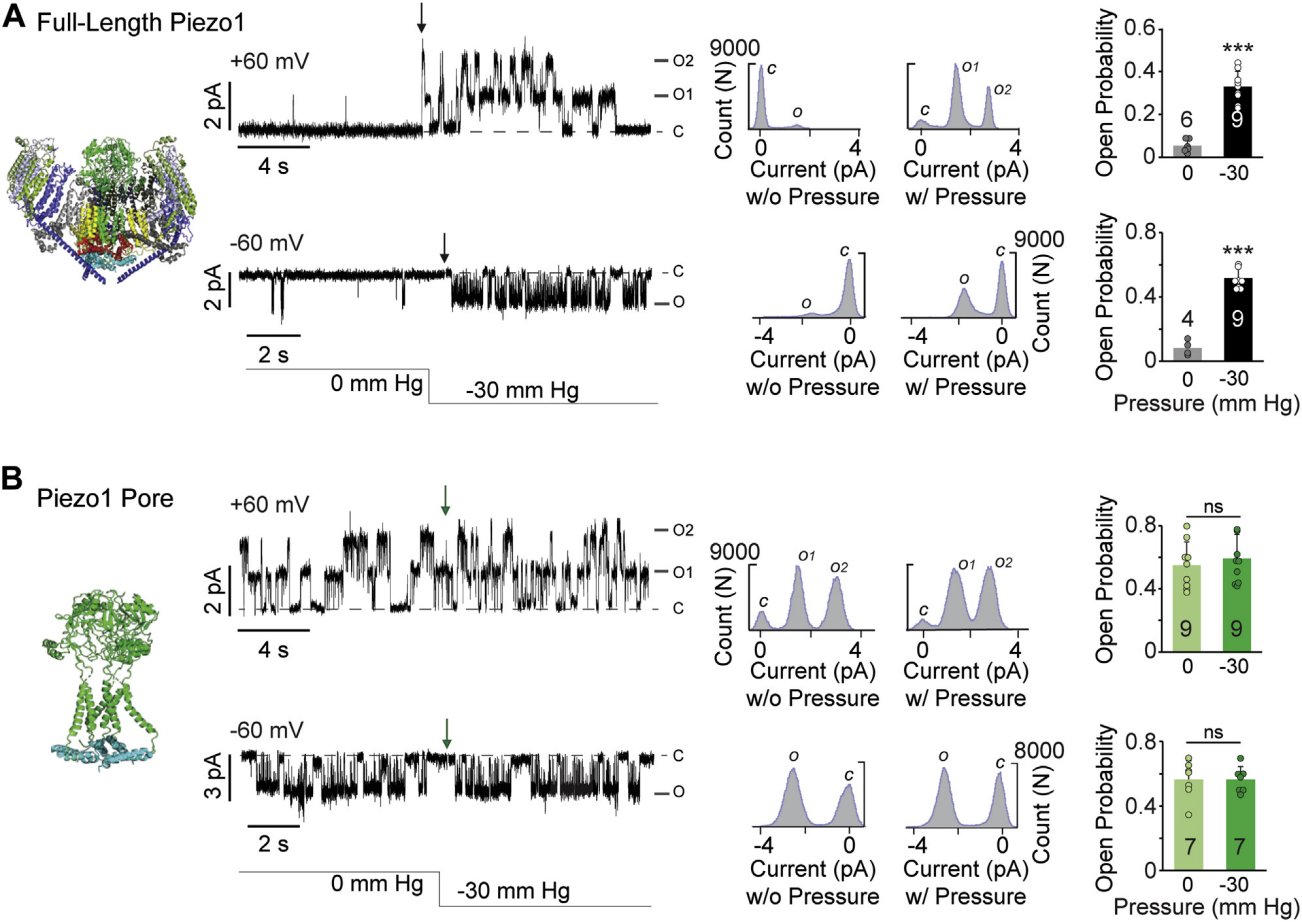
**The pressure-dependent open probability of full-length Piezo1 and pore domain.***A*, *left to right*, full-length Piezo1 EM structure. Single-channel currents at ± 60 mV at 0 and −30 mm Hg in cell-attached mode, arrow indicates application of negative pressure, *O* and *C* denote open and closed state of the channel. All-point current histograms at ± 60 mV with and without pressure application. Normalized open probability bar graphs at +60 mV (*top*) and −60 mV (*bottom*). The open probability is presented as Mean ± Standard Deviation, two-tailed *t*-test: ∗∗∗*p* < 0.0005. *B*, *left to right*, Piezo1 pore domain E2172 representation based on full-length Piezo1 structure. Single-channel currents at ±60 mV acquired at 0 and −30 mm Hg in cell-attached mode, arrow indicates application of negative pressure, *O* and *C* denote open and closed state of the channel. All-point current histograms at ±60 mV with and without pressure. Normalized open probability bar graphs at +60 mV (*top*) and −60 mV (*bottom*). The open probability is presented as Mean ± Standard Deviation, one-tailed *t*-test: ns *p* > 0.06 at +60 mV, *p* > 0.98 at −60 mV.

First, the activity of full-length Piezo1 and pore domain was recorded without any applied pressure, followed by the immediate application of a negative pressure while keeping a constant voltage pulse of +60 or −60 mV (Fig. 5, *A*–*B*). As expected, a burst of Piezo1 channel activity was observed only in the presence of pressure application. Additionally, we were able to quantify the sporadic and spontaneous activity of Piezo1 at +60 mV in the absence of pressure (in n = 6 out of 9 experiments) (Fig. 5*A* upper panel). The normalized open probability of full-length Piezo1 without pressure was NPo = 0.05 ± 0.03 and with pressure NPo = 0.33 ± 0.07. Conversely, the number of channels and open probability of pore domain remained the same before and after pressure application NPo = 0.55 ± 0.15 *versus* NPo = 0.59 ± 0.15 (Fig. 5*B* upper panel).

Next, the pressure-dependent activity of full-length Piezo1 and pore domain was compared at negative potentials (Fig. 5, *A*–*B* lower panels). As expected, Piezo1 exhibited channel activity only under applied stimulus, while pore domain was insensitive to pressure. The single-channel open probability of Piezo1 without pressure was significantly lower (NPo = 0.08 ± 0.04) compared with when pressure was applied (NPo = 0.52 ± 0.06). There was no effect of pressure on Piezo1 single-channel current amplitude (Fig. 5*A*) when compared with and analyzed by a two-tailed *t*-test (*p* > 0.05). The pore domain was fully functional without the pressure stimulus, and the statistical analysis showed no significant difference in open probability when pressure was applied (NPo = 0.57 ± 0.11 *versus* 0.56 ± 0.08) (Fig. 5*B* lower panel). Similarly, S1953 truncated mutation did not exhibit pressure sensitivity when tested at ± 60 mV (NPo= 0.42 ± 0.18 *versus* 0.6 ± 0.12 at −60 mV and NPo= 0.44 ± 0.08 *versus* 0.4 ± 0.11 at 60 mV, data shown in source file).

**Figure 6:**
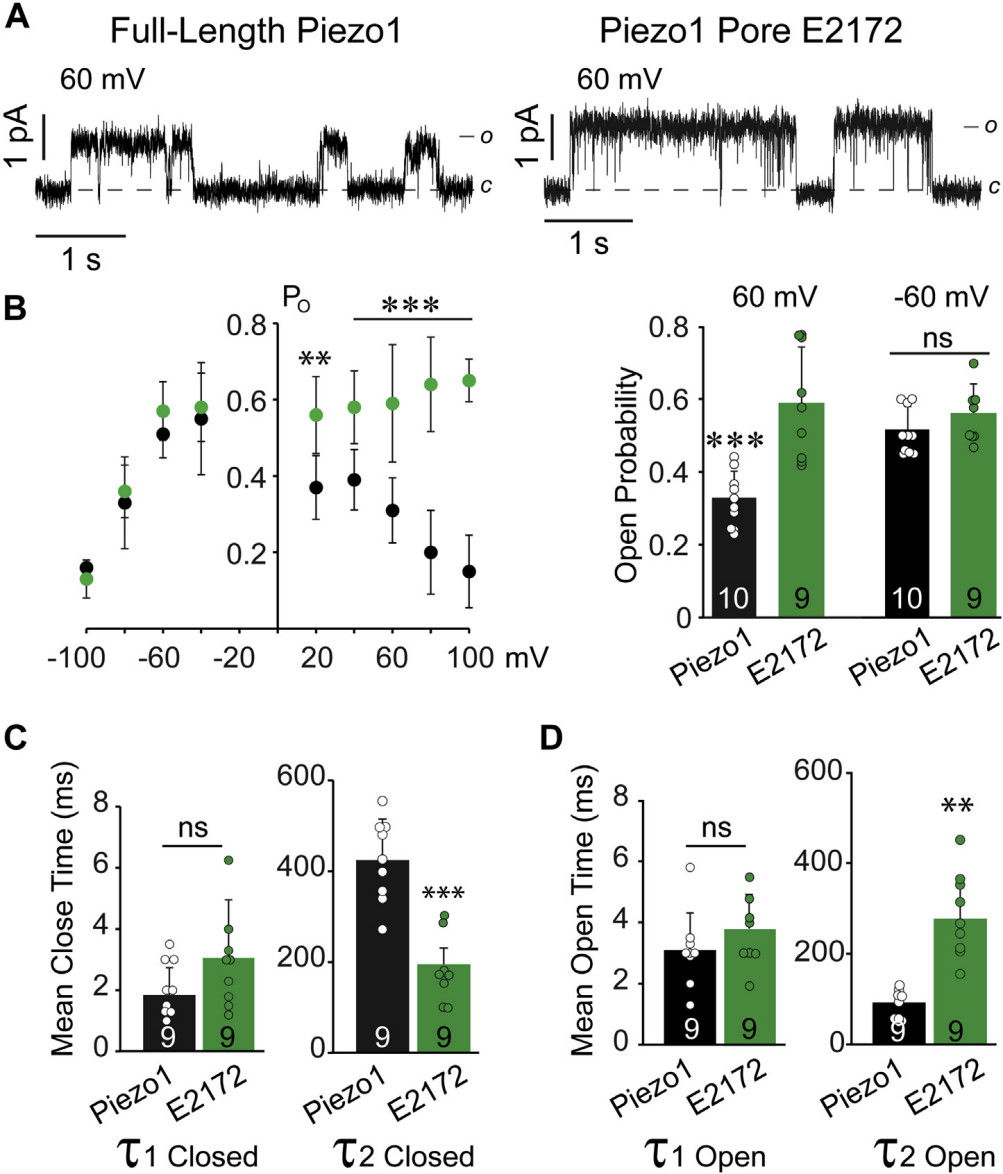
**Single-channel kinetic analysis of full-length Piezo1 and pore domain.***A*, representative single-channel traces of full-length Piezo1 and its pore domain at +60 mV highlighting the differences in open probability. *B*, normalized open probability (NPo) of full-length Piezo1 (*black*) and pore domain (*green*) (n ≥ 5) acquired at various potentials (*left*) and the NPo comparison at ± 60 mV. *C*, comparison of mean closed time constants (τ_1 Closed_ and τ_2 Closed_) at +60 mV between full-length Piezo1 and the pore domain. *D*, comparison of mean open time constants (τ_1 Open_ and τ_2 Open_) at +60 mV between full-length Piezo1 and the pore domain. The data is presented as Mean ± Standard Deviation, *t*-test: where ∗∗∗*p* < 0.0005, ∗∗*p* < 0.005 and ns *p* > 0.11.

The comparison of open probability at various applied potentials showed voltage dependence in full-length channels, but not in the pore domain (Fig. 6, *A*–*B*). The heightened NPo of the Piezo1 pore domain at positive potentials may result from an increase in the mean open time and/or decreased mean close time distributions. To address this question, we analyzed the distribution of closed and open dwell times at +60 mV where the significant increase was observed in the open probability of the pore domain (Fig. 6*B*). Focusing first on the role of the full-length Piezo1 (including the sensing region) in favoring the channel’s closed state, we find that the closed dwell times are fitted by two component probability density function, τ_1 Closed_ (short-lived closed time constant) and τ_2 Closed_ (longer-lived close time constant). A significant increase in τ_2 Closed_ was observed for full-length Piezo1 as compared with the pore domain; 423 ± 90 ms, (Full length Piezo1) and 192 ± 36 ms, (Piezo1 Pore domain). Whereas no significant difference was observed in τ_1 Closed_ 1.8 ± 0.9 ms *versus* 3.1 ± 1.9 for full-length Piezo1 and pore domain, respectively (Fig. 6*C*).

The open dwell time distributions are also fitted with two time constants, τ_1 Open_ (short-lived open time constant) and τ_2 Open_ (long-lived open time constant). Consistent with τ_1 Closed_ no significant difference was observed in τ_1 Open_, while Piezo1 full-length exhibit decreased τ_2 Open_ = 92 ± 30 ms as compared with the pore domain τ_2 Open_ = 276 ± 80 ms (Fig. 6*D*). The data suggest that in the absence of a sensing module, the pore module was constitutively open at the expense of increased mean open time (τ_2 Open_) and decreased mean closed time (τ_2 Closed_), especially at positive potentials.

## Discussion

### Effect of structurally identified elements on ion permeation

The cryo-EM structure and chimeric studies provided hints that the Piezo1 pore exists within the last ∼450 amino acids (33, 38). The large size of the protein proved to be challenging in delineating the exact amino acid sequence of the pore domain. By conducting serial deletions and systematic functional studies, we identified the pore domain that recapitulates the permeation hallmarks of the full-length Piezo1 (slope conductance and pharmacology), but did not preserve any pressure sensitivity. Furthermore, the presence of divalent ions decreased the full-length Piezo1 rectification, but not that of the pore domain (Figs. 3*A* and 4). These interesting results point toward a divalent (Ca^2+^ or Mg^2+^) binding site that is outside the permeation pathway. The significant deletion of Piezo1 to identify the pore domain must have interrupted interactions between different regions of the protein and the pore domain. The previously identified portals and plugs (39) were abolished, especially on the intracellular side: i) lateral plug gate from residue 1396 to 1405, ii) latch region from residue 1406 to 1420, central plug from residue 1382 to 1395 amino acids, among others. These plugs and portals are suggested to participate in permeation properties, and indeed the deletions of plugs increased Piezo1 single-channel conductance. The construction of our pore domain and truncated proteins (starting from residues E2172, G2124, and S1953) led us to indirectly delete the interaction of portals and plugs, which likely lead to an increased outward rectification at higher potentials.

### Mechanical properties of truncated proteins

The single-channel data suggested that the pore domain and deletion mutants do not harbor pressure sensitivity when tested at positive and negative potentials, and the channels were fully capable of conducting ions without applied mechanical stimulus (Figs. 5 and 6). Piezo Repeat A and the anchor regions are not required for pressure sensitivity. Therefore, the sensing module must be contained within Piezo1 residues 1 to 1952. These data are reminiscent of the voltage-gated potassium channels, where the pore module was conductive without the sensing module, and one of the suggestive roles of the sensors was to keep the channel in a closed confirmation (40). More work remains to be conducted to identify and narrow down the key sensing domain(s) within the ∼1950 amino acid sequence. The beam helix is a promising candidate for inclusion in the sensing domain due to the identified point mutations that alters mechanosensitive gating. However, deletion of distal loops connecting transmembrane segments 15 to 16 and 19 to 20 also alter mechanical gating of Piezo1 (34). It is conceivable that Piezo1 might not have a canonical “sensing” sequence with a well-defined sensing domain (41), such as the S4 segment, with arginine residues in voltage-gated potassium channels.

Modularity appears to be a key feature of ion channels. Separate modules have the propensity to operate outside their native context, while preserving physiological footprints. The concept of modularity was explicitly proposed in 1990 (42) and the first experiments performed by Montal *et al.* (43), to identify the pore module of voltage-gated ion channels by constructing chimeras between the Kv1.1 sensing module and Shaker’s pore module, and vice versa. This idea opens the door to study the pore module of ion channels in isolation, as seen for two pore domain potassium channels (44), voltage-gated potassium (28, 40) and sodium channels (29). The identification of a structurally recognized functional pore module of mechanosensitive Piezo1 has vast implications for protein engineering, rational drug design, and small-molecule screening. Importantly, most of the Piezo1 disease-causing point mutations (15) are in the pore domain of the channel and the last six transmembrane segments, making this specific part of the protein a key pharmacological target.

## Experimental procedures

### Cell culture and transient transfection

HEK293T cells with genomic deletion of Piezo1 (HEK293T^ΔP1^) cells were cultured in Dulbecco’s modified Eagle’s medium (DMEM) supplemented with 10% fetal bovine serum (FBS) and 1% penicillin/streptomycin (Gibco). Cells were plated on Poly-D-lysine (Gibco) coated coverslips 6 to 20 h before transfection, in antibiotic-free medium. Transient transfection was performed using Lipofectamine 3000 (ThermoFisher, Waltham, MA) according to the manufacturer’s instructions.

### Molecular biology

The full-length mouse Piezo1 gene was synthesized using GenScript’s Clone EZ service. Subsequent deletion clones for Piezo1 starting from S1953, G2124, and E2172 were generated from this clone, also using GenScript’s Clone EZ. All nucleotide sequences were sequence verified after every maxi prep DNA isolation.

### Electrophysiology

Transfected HEK293T^ΔP1^ cells were visualized using Nikon eclipse Ti2 microscope and C11440 Orca-Flash 4.0 LT digital camera (Hamamatsu). Cell-attached or excised inside-out recordings of mechano-activated currents in HEK293T ^ΔP1^ cells expressing the full-length Piezo1 and truncated proteins were performed using experimental setup based on an Axopatch 200B amplifier and Digidata 1550B digitizer (Molecular Devices). Currents were acquired with pClamp 10.7 software and were recorded at a sampling frequency of 10 kHz. Recording patch pipettes of borosilicate glass were pulled and fire-polished to a tip resistance of 4 to 6 MΩ. The bath solution contained (in mM): 140 KCl (or 140 NaCl as described), 10 HEPES, 1 MgCl_2_, 10 glucose, pH 7.3 (pH adjusted with KOH). The pipette solution contained (in mM): 130 NaCl, 5 KCl, 10 HEPES, 10 TEA-Cl, 1 CaCl_2_, 1 MgCl_2_, pH 7.3 (pH adjusted with NaOH), for divalent-free solution CaCl_2_ and MgCl_2_ inside the pipette were substituted with 0.25 mM EGTA. The bath electrode was grounded, while the pipette electrode was active, such that at positive applied potential cations moved from pipette to bath, and vice versa. Mechanical stimulation was applied *via* recording pipette using a high-speed pressure clamp system (ALA Scientific Instruments). Currents were recorded at voltages ranging from −100 mV to 100 mV. Single-channel events are shown as down or up deflections at a given voltage representing outward or inward current, respectively. This discrepancy in current flow (*i.e.*, outward currents at negative potentials) is due to its inside-out and cell-attached patch configurations. Nevertheless, the outward currents were larger than the inward currents. The single-channel data was analyzed using Clampfit 10.7.

### Analysis and statistics

All-point current histograms of the single-channel data (ranging from 5 to 20 s stretch) were constructed in Clampfit. The single-channel current values were extracted after fitting the Gaussian function to the data. I-V plots were presented as Mean ± Standard Deviation. Each data point is at least an average of 5 or at most 17 individual recordings. Open probability was calculated exclusively from the records, where at least 10 s of data was recorded both before and after pressure application. The number of channels in the patch is determined by fitting a Gaussian curve to all-point current histograms. At least a 15 to 20 s stretch was analyzed to observe for consecutive channel openings. The equally spaced Gaussian peaks determined the presence of one, two, or three channels (example Fig. 5, *A*–*B* upper panels). To obtain mean open (τ _Open_) and mean close (τ _Closed_) time constants, exponential log probability density function was fitted to mean open and closed dwell time distributions. Group data (conductance bar graphs) are presented as Mean ± Standard Deviation and ∗ *p* < 0.05; ∗∗*p* < 0.05, ∗∗∗*p* < 0.005, not significant (ns) *p* > 0.05 Unpaired two-tailed *t*-tests were used for comparison between the two groups, as indicated. For multiple group comparisons, a one-way ANOVA with Bonferroni correction was performed (source files).

## Data availability

All data pertinent to this work are contained within this article and also available upon request. For requests, please contact Ruhma Syeda at Ruhma.syeda@utsouthwestern.edu

## Conflict of interest

The authors declare no conflicts of interest with the contents of this article.

## Supporting information

JBC_JBC/2020/015905_0.xlsx

JBC_JBC/2020/015905_1.xlsx

JBC_JBC/2020/015905_2.xlsx

JBC_JBC/2020/015905_3.xlsx

JBC_JBC/2020/015905_4.xlsx
